# 

**Published:** 1890-04

**Authors:** 


### CONTENTS.

***EDITORIALS.***

PAGE

The International Congress, . . . . . . . . . . . . 145

The Value of Trivial Symptoms, . . . . . . . . . . 146

***MISCELLANEOUS.***

What Constitutes a Homœopathic Physician. P. P. Wells, M. D., . 148

Clinical Reports in their Relation to Homœopathy. Clarence Willard Butler, M. D., . . . . . . . . . . . 150

The International Homœopathic Congress of Paris. Translated by Walter M. James, M. D., . . . . . . . . . . 155

The International Homœopathic Congress of Paris, August 21st, 22d, and 23d, 1889. Translated by E. A. P., . . . . . . . 157

The Question of the Dose, . . . . . . . . . . 159

The Germ Theory and its Relation to Homœopathy, . . . 161

The Homœopathic Materia Medica, . . . . . . . 163

A Plan to Reform the Materia Medica, . . . . . . 164

A Plea for the Cyclopædia of Drug Pathogenesy, . . . 165

Electro-Homœopathy, . . . . . . . . . . . 167

Polypharmacy, or Mixed Prescriptions, . . . . . . 168

Progressive Locomotor Ataxia, . . . . . . . . 169

Insomnia and Neuralgia, . . . . . . . . . . 171

Iritis and Irido-Choroiditis, . . . . . . . . . 171

Diphtheria and Croup, . . . . . . . . . . . 172

Cyanide of Mercury in Diphtheria, . . . . . . . 173

Paralysis Agitans, . . . . . . . . . . . . 176

Polypharmacy and the Single Remedy, . . . . . . 177

Mixed Medicines in one Prescription Absurd, . . . . 179

The Three Essential Precepts of Homœopathic Therapeutics, . 179

Denunciation of Mixed Prescriptions, . . . . . . 180

A Plea for Alternation, . . . . . . . . . . 181

Dr. Conan's Defense of Mixed Prescriptions, . . . . 181

A Declaration for Pure Homœopathy, . . . . . . . 182

Repudiation of Electro-Homœopathy, . . . . . . . 183

A Clinical Case. Wm. Steinrauf, M. D., . . . . . . . 184

Clinical Cases. F. L. Griffith, M. D., . . . . . . . . 185

The Ward's Island "Difficulty" in New York, . . . . . . 188

***BOOK NOTICES.***

Homœopathy. By S. Mills Fowler, M. D., . . . . . . . 190

Spinal Concussion. By S. V. Clevenger, M. D., . . . . . 190

***NOTES AND NOTICES.***

Corrections—Dr. William S. Gee—Removals—Cholera at Bagdad—American Institute of Homœopathy—Grace Hospital—Extract from a Letter of Thackeray—To Biological Students—The Doctor Imposed on—New York State Medical Society—Fun for Doctors, . . . . . . . . . . . . . . 191

